# Interleukin‐10 promoter polymorphisms and haplotypes in patients with Guillain–Barré syndrome

**DOI:** 10.1002/acn3.51939

**Published:** 2023-11-13

**Authors:** Shoma Hayat, Asaduzzaman Asad, Moriam Akter Munni, Md. Abu Jaher Nayeem, Md. Golam Mostafa, Israt Jahan, Md. Zakir Hossain Howlader, Quazi Deen Mohammad, Zhahirul Islam

**Affiliations:** ^1^ Laboratory of Gut‐Brain Axis Infectious Diseases Division (IDD), icddr,b Dhaka Bangladesh; ^2^ Department of Biochemistry and Molecular Biology University of Dhaka Dhaka Bangladesh; ^3^ National Institute of Neurosciences and Hospital Dhaka Bangladesh

## Abstract

**Objective:**

Interleukin‐10 (IL‐10) is a multifunctional cytokine that exerts both pro‐ and anti‐inflammatory effects on the immune system as well as in the pathogenesis of Guillain–Barré syndrome (GBS). We investigated whether the three common polymorphisms ‐1082 G/A(rs1800896), ‐819 C/T(rs1800871), and ‐592 C/A(rs1800872) in the promoter region of IL‐10 have any influence on the susceptibility, severity, and clinical outcome of GBS.

**Methods:**

IL‐10 promoter polymorphisms were investigated in 152 patients with GBS and 152 healthy controls from Bangladesh using polymerase chain reaction and restriction fragment length polymorphism (PCR‐RFLP), and allele‐specific oligonucleotide‐PCR (ASO‐PCR). Haplotype patterns and frequencies were analyzed using Heatmaply R‐package, chi‐square, and Fisher's exact test. The serum level of IL‐10 was measured through enzyme‐linked immunosorbent assays. *p*‐values < 0.05 were considered statistically significant.

**Results:**

IL‐10 promoter polymorphisms ‐1082 G/A, ‐819 C/T, and ‐592 C/A were not associated with GBS susceptibility. The homozygous ‐819 TT genotype showed a tendency with susceptibility (*p* = 0.029; *p*c = 0.08) and was prevalent in axonal variants of GBS compared to demyelinating subtypes and controls (*p* = 0.042, OR = 8.67, 95% CI = 1.03–72.97; *p*c = 0.123 and *p* = 0.005, OR = 4.2, 95% CI = 1.55–11.40; *p*c = 0.015, respectively). Haplotype analysis revealed 19 patterns of genotypes and high IL‐10 expression haplotype combinations (GCC/GTA, GCC/ATA, and GCC/GCA) may have influence on disease severity (*p* = 0.026; *p*c = 0.078). Serum expression of IL‐10 was elevated in GBS patients ([GBS, 12.16 ± 45.71] vs. [HC, 0.65 ± 5.17] pg/mL; *p* = 0.0027) and varied with disease severity ([severe‐GBS, 15.25 ± 51.72] vs. [mild‐GBS, 3.59 ± 19.79] pg/mL, *p* = 0.046).

**Interpretation:**

The ‐819 TT genotypes influence axonal GBS, and high frequency of IL‐10 expression haplotype combination with elevated serum IL‐10 may play an important role in disease severity.

## Introduction

Guillain–Barré syndrome (GBS) is an autoimmune‐mediated disorder affecting the axons and myelin sheath of the peripheral nervous system (PNS) with high clinical disability.^1^ Presently, GBS is considered as an excellent paradigm of molecular mimicry in which lipo‐oligosaccharide in the outer core structure of the infectious agents induce the formation of cross‐reactive antibodies against host nerve gangliosides.^2^ These pathogenic antibodies result in an aberrant immune system and subsequent peripheral nerve damage.^3^, ^4^, ^5^ Based on recent evidences, molecular mimicry alone is not enough to explain the etiology of GBS. The immune response to host susceptibility may also play an essential role in the induction of the disease.^6^, ^7^ Strain properties and host properties are both crucial in determining the risk of developing GBS.^8^ Host factors and their genetic predisposition to GBS is very important to decipher their role in GBS pathogenesis as well as disease progression and severity.^9^, ^10^, ^11^ Therefore, the serum IL‐10 level and polymorphisms in it may be potential decider here.

IL‐10 is an important cytokine in the regulation of inflammatory and immune responses and has been implicated in autoimmunity.^12^ This cytokine, produced by B cells, T cells, and macrophages, is also considered as the “cytokine synthesis inhibitory factor” (CSIF) that inhibits the release of T‐helper (Th) 1‐type cytokines such as tumor necrosis factor alpha (TNF‐α), interferon gamma (IFN‐γ), and IL‐2,^13^, ^14^, ^15^, ^16^ and downregulates the expression of major histocompatibility complex (MHC) class II on macrophages.^17^ IL‐10 may also contribute to pro‐inflammatory actions of the immune system, such as the activation of B cells, along with the production of auto‐antibodies and inhibition of T cell apoptosis. All these effects are considered very important in the pathogenesis of GBS.^18^

The IL‐10 gene is chromosomally located at 1q31‐1q32,^19^, ^20^ and the production of this cytokine is strongly influenced by genetic factors.^21^ Several polymorphic sites have been described in the promoter region of IL‐10 gene including the bi‐allelic polymorphisms at ‐1082 G/A (rs1800896), ‐819 C/T (rs1800871) and ‐592 C/A (rs1800872) locus of the gene's transcriptional start site which are most common and important in the pathogenesis of autoimmune diseases.

Several studies were performed to observe whether these single‐nucleotide polymorphisms (SNPs) of IL‐10 gene encoding macrophage mediators are responsible for the severity and susceptibility of GBS.^18^, ^22^ Press et al. showed that high levels of IL‐10‐secreting blood mononuclear cells (MNCs) correlated with the serum levels of anti‐ganglioside antibodies and axonal damage suggesting the upregulation of IL‐10 expression in the early phase of GBS development.^1^, ^23^ In a Norwegian population, ‐592 CC and ‐819 CC genotypes were associated with increased IL‐10 response in GBS.^18^ One Dutch study reported no associations between the single‐nucleotide polymorphisms (SNPs) in IL‐10 promoter region and disease susceptibility or subgroups.^22^ However, very few data are available regarding IL‐10 polymorphism from low‐income countries. Therefore, we intended to investigate the distribution of IL‐10 promoter polymorphisms and their influence on disease susceptibility, severity, and prognosis in patients with GBS from a well‐documented cohort of Bangladesh.

## Materials and Methods

### Study subjects

This study included 152 patients with GBS (103 males and 49 females; median age, 29 years [interquartile range, 17–42 years]) and 152 healthy individuals from Bangladesh (78 males and 74 females; median age, 35 years [interquartile range, 28–40 years]). Healthy controls were genetically unrelated to the patients and ethnically matched with no history of previous GBS or other neurological disorders or comorbidities. Healthy controls were enrolled during patient enrolment period. Patients were enrolled from Dhaka Medical College and Hospital (DMCH) after the onset of neuropathic symptoms that fulfilled the diagnostic (National Institute of Neurological Disorders and Stroke [NINDS]) criteria for GBS as described by Asbury and Cornblath.^24^ Enrolled patients with GBS received neither intravenous immunoglobulin (IVIg) nor underwent for plasma exchange therapy; all patients were supportively cared. Written informed consent was obtained from each study subject before clinical examination, specimen collection, and data collection. This study was reviewed and approved by the Institutional Review Board (IRB) and ethical committees of icddr,b, Dhaka, Bangladesh. Data were collected on the basis of age, sex, antecedent events, detailed neurological signs and symptoms, treatment, days to nadir, complications, duration of admission, GBS disability score (GBS‐DS),^25^ and the Medical Research Council (MRC)^26^ sum score at four standard points (entry, 2 weeks, 4 weeks, and 6 months after enrollment). Both the GBS‐DS and MRC sum score indicated the severity of disease. Patients with an MRC sum score at nadir of <40 were defined as severely affected, and patients with a score of 40–60 were defined as mildly affected.^22^ Patients with GBS‐DS of 0, 1, and 2 (independent walking) within 6 months represented good outcome and patients with GBS‐DS of 3, 4, 5, and 6 (unable to walk or death) represented poor outcome.^22^, ^25^

### Detection of serum expression of IL‐10 in patients with GBS and healthy controls

Serum levels of IL‐10 were measured in 151 patients with GBS (111 severely affected and 40 mildly affected) and in 151 age‐ and sex‐matched healthy controls by enzyme‐linked immunosorbent assays (ELISAs) technology using human IL‐10 ELISA kits (human IL‐10: Thermo Scientific EHIL10). The ELISA assays were performed on serum samples in duplicate collected during the entry of study subject enrolment following the manufacturer's instructions. The results of the ELISA assays were expressed as picograms of IL‐10 per milliliter (pg/mL).

### Detection of *Campylobacter jejuni* infection and anti‐ganglioside antibodies

Serum samples were separated from pretreated blood collected from 152 patients with GBS for serological assay of recent *C. jejuni* infection and determination of nerve anti‐ganglioside antibodies (e.g., GM1). Serological assays were conducted in duplicate using previously described indirect enzyme‐linked immunosorbent assays (ELISAs) techniques to detect IgG, IgM and IgA antibodies against *C. jejuni* and auto‐antibodies against GM1, GD1a and GQ1b.^27^, ^28^, ^29^

### Genomic DNA isolation and detection of IL‐10 polymorphisms

Genomic DNA of 304 study subjects were extracted from whole blood using a QIAamp® DNA Blood Midi Kit (100; Qiagen, Hilden, Germany) according to the manufacturer's protocol. The DNA samples were dissolved in 1X TE‐buffer (10 mM Tris‐Cl, pH 8.0 & 1 mM EDTA) and eventually diluted with Milli‐Q water to a final concentration of 10 ng/μL and stored at −20°C for polymorphism study. IL‐10 SNPs including ‐1082 G/A (rs1800896) and ‐592 C/A (rs1800872) were determined by polymerase chain reaction‐restriction fragment length polymorphism (PCR‐RFLP) assay consisting of an initial PCR followed by specific restriction endonuclease *Mnll* and *Rsal digestion*, respectively.^20^ Allele‐specific oligonucleotide‐polymerase chain reaction (ASO‐PCR) assay was performed to detect ‐819 C/T(rs1800871) polymorphisms. For ASO‐PCR, primers were designed using NCBI public database and OligoAnanlyzer 3.1.^30^, ^31^ Details of the primers and enzymes used in the study for detection of SNPs were presented in Table S1. Master mix (25 μL) was prepared containing 10 ng of genomic DNA, 10 pmol of each specific primer, 0.1 mM dNTPs (Promega), 1 U of GoTaq® Flexi DNA Polymerase (Promega), 5× Green GoTaq® Flexi Buffer, 25 mM MgCl_2_ and Milli‐Q to perform PCR analysis. After digestions, the digested products and PCR products were visualized on 2% agarose gels using a Molecular Imager® Gel Doc™ XR + system (Bio‐Rad Laboratories Inc, USA).

### Statistical analysis

Genotypes (combination of alleles of a given SNP) and allele frequencies were analyzed using chi‐square (*χ* ^2^) test and Fisher's exact test with Yates correction. Hardy–Weinberg equilibrium was analyzed for healthy individuals using chi‐square (*χ* ^2^) test. For a value less than 5 in any cell of the 2 × 2 table, Yates correction was performed and considered significant at a probability level (*p*) of <0.05. The Bonferroni method was used to correct the *p*‐values for multiple comparisons where each *p*‐value was multiplied by the number of comparisons and represented as *p*c (*p*c, *p* corrected). The results were represented as odds ratio (OR) showing 95% CI as well. Haplotypes and allele frequencies were estimated by simple gene counting, and the data were processed in Microsoft Excel 2007. Haplotype patterns, graphical representation, and frequencies were analyzed using the Heatmaply v1.3.0 package of R statistics v4.0.5, and their associations with GBS susceptibility and subgroups were assessed using logistic regression. The data of serum levels of IL‐10 were expressed as mean with standard deviation (Mean ± SD). The differences in the serum concentrations of IL‐10 (pg/mL) between healthy controls and GBS or subgroups of GBS were analyzed using the unpaired *t*‐test with Welsh's correction. *p*‐value < 0.05 was considered as level of significance. Statistical analyses were performed using the GraphPad Prism (version 5.01, GraphPad Software, Inc. La Jolla, CA 92037, USA) and SPSS (20.0 version, Chicago, IL, USA) computer software programs.

## Results

### Clinical and serological characteristics

**Figure 1:**
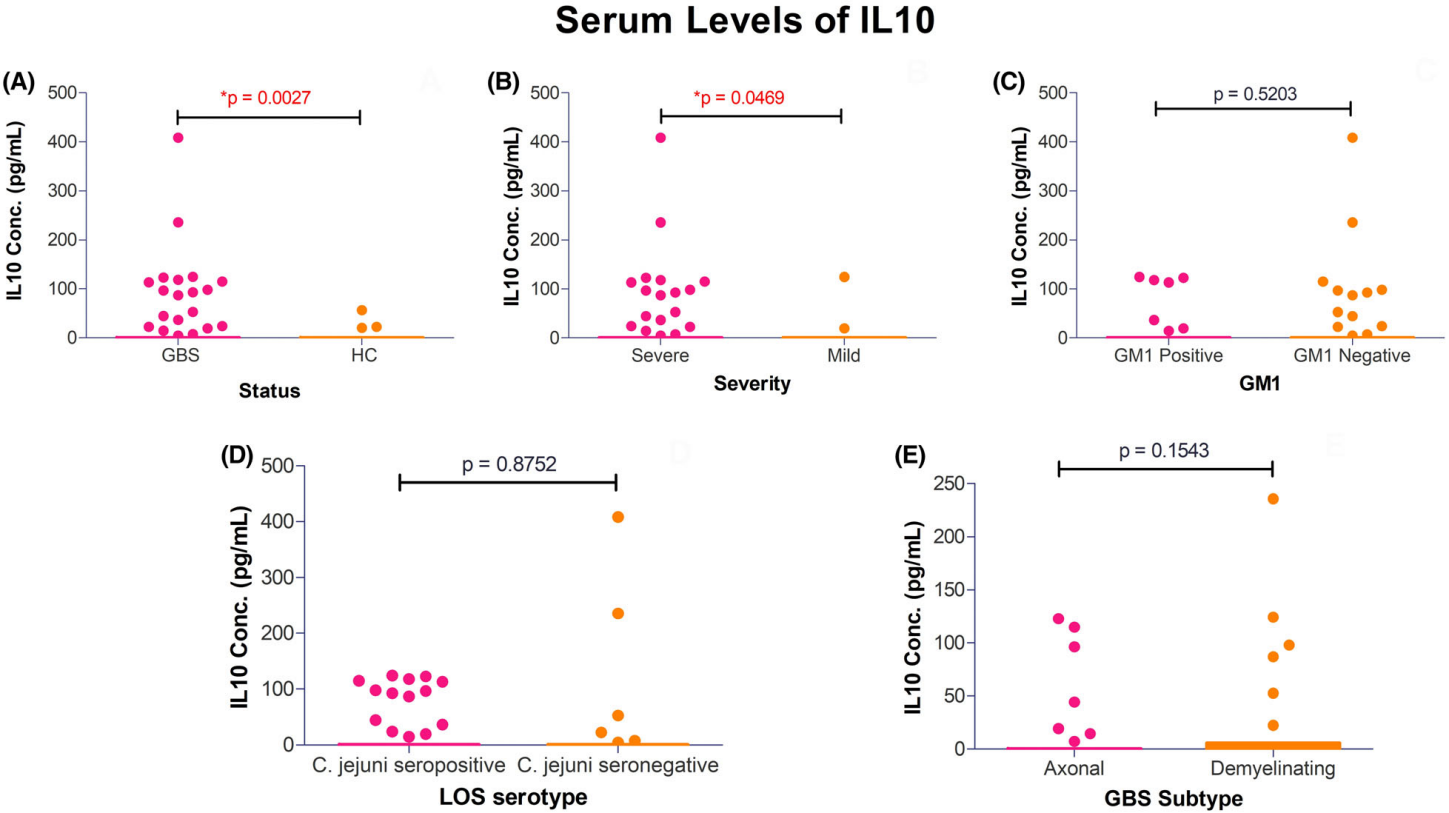
Serum levels of IL‐10 (pg/mL) in study population. Whiskers (Tukey) plot on (A) Serum expression levels in GBS patients vs. healthy controls. (B) Serum levels in severely affected patient vs. mildly affected patients. (C) Serum levels in anti‐GM1 antibody (Ab) positive and anti‐GM1 Ab negative patients. (D) Serum levels in LOS seropositive and LOS seronegative patients. (E) Serum levels in axonal vs. demyelinating subtypes of GBS. Significance of mean difference (*p*‐value) was observed from unpaired *t*‐test with Welch's correction method. *Statistically significant mean differences.

Among the patients, 86% (130/152) individuals had antecedent events of infection; 55% (71/130) with diarrhea, 18% (24/130) with respiratory infection and 13% (17/130) with fever and 14% (18/130) with Varicella‐zoster, measles, flu‐like infection, and other unknown preceding illnesses. Electrophysiological studies on 68% (104/152) patients with GBS revealed, 57% (59/104) were axonal variants of GBS (55 acute motor axonal neuropathy [AMAN] and 4 acute motor and sensory axonal neuropathy [AMSAN]), 26% (27/104) were demyelinating type (acute inflammatory demyelinating polyradiculoneuropathy [AIDP]), and 17% (18/104) were unclassified GBS cases with inexcitable nerves or equivocal findings. Among the patient with GBS, 38% (58/152) had anti‐GM1 antibody (Ab) positivity, 15% (23/152) had anti‐GD1a Ab positivity, and 9% (14/152) anti‐GQ1b Ab had seropositivity, respectably (Table 1). Serum levels of IL‐10 were estimated in patients with GBS (0.0–408.6 pg/mL) and in healthy control (0.0–56.40 pg/mL). Serum levels were elevated in patients with GBS compared to healthy control (Mean [GBS], 12.16 ± 45.71 pg/mL vs. mean [Healthy control], 0.65 ± 5.17 pg/mL; *p* = 0.0027; Fig. 1A).

**Table 1:** Demographic and clinic‐serological features of patients with GBS (*n* = 152).

Characteristics	Number of patients, *n* = 152 (%)
Sex	
Male/Female	103/49 (68/32)
Age	
Median (IQR, full range)	29 (17–42)
Preceding illness	*n* = 130/152 (86)
Diarrhea	71/130 (55)
Respiratory tract infections	24/130 (18)
Fever	17/130 (13)
Other illness	18/130 (14)
Electrophysiological classification, *n* = 104	*n* = 104
Axonal type	59/104 (57)
Demyelinating type	27/104 (26)
Unclassified	18/104 (17)
Severity based on MRC sum score (at entry), *n* = 152	
Severely affected patients (MRC < 40)	111/152 (73)
Mildly affected patients (MRC 40–60)	41/152 (27)
Serological characteristics, *n* = 152	
Anti‐GM1 Ab positivity	58/152 (38)
Anti‐GD1a Ab positivity	23/152 (15)
Anti‐GD1b Ab positivity	14/152 (9)
Disease prognosis based on GBS‐DS at 6 months, *n* = 152	
Good outcome	96/152 (63)
Poor outcome	56/152 (37)

Ab, antibody; GBS, Guillain–Barré syndrome; GBS‐DS, GBS disability score; IQR, interquartile range; MRC, Medical Research Council.

### Distribution of haplotype patterns in 3 loci of IL‐10 promoter polymorphisms

**Figure 2:**
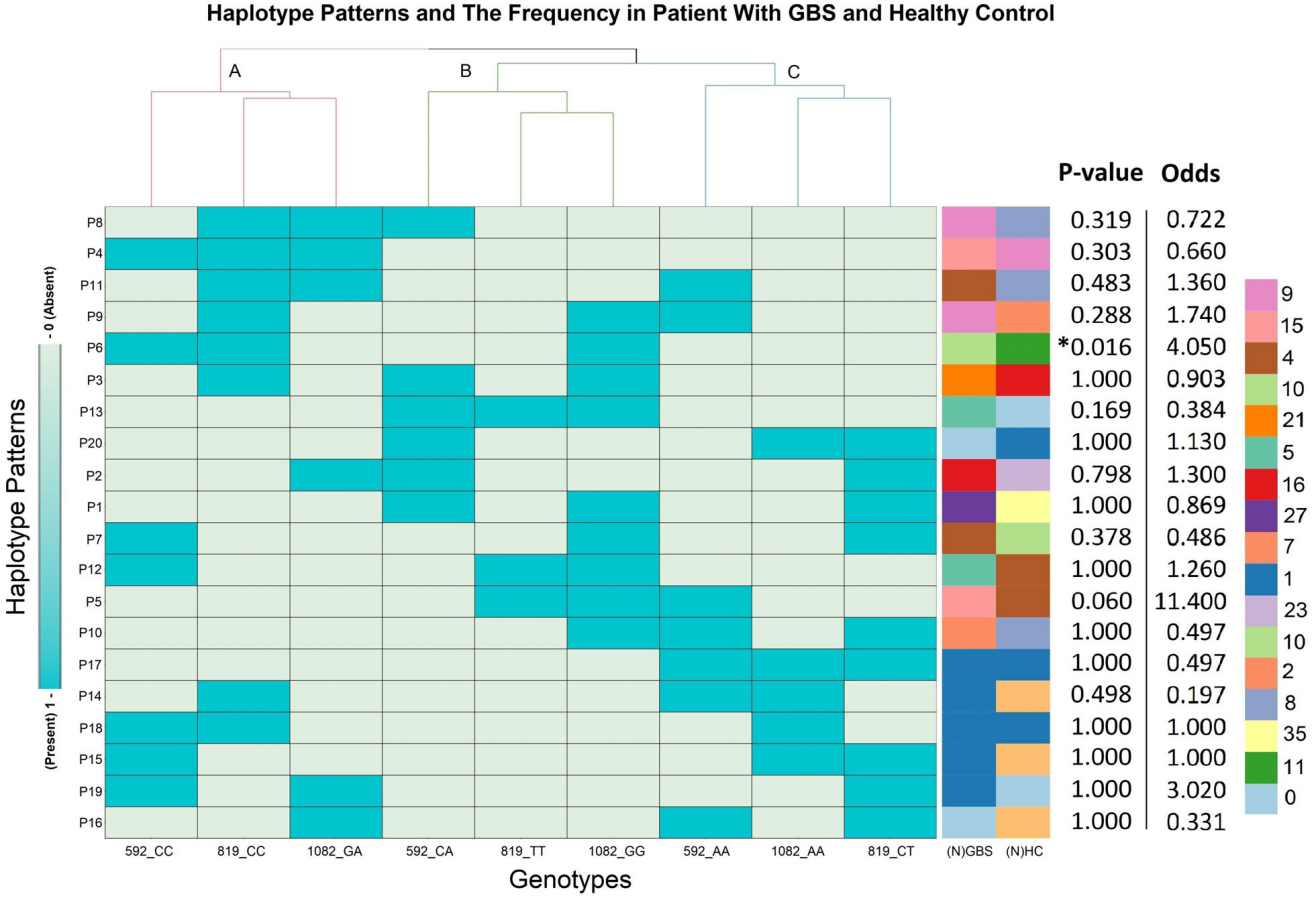
Haplotype patterns for three loci at ‐1082 G/A, ‐819 C/T, and ‐592 C/A position of interleukin‐10 promoter region. Nineteen haplotype patterns were observed for corresponding SNPs of interleukin‐10 promoter region in study subjects. Separately clustered multicolor bars showed the frequencies of haplotypes in patients with GBS [(N)GBS] and healthy control [(N)HC]. Left and right scale‐bar represented the presence (cyan color)/absence (pale blue) of the genotypes and frequencies with the color blocks, respectively. Column wise cladogram (A–C) showed clustering with higher possibility of co‐occurrence of allele types and row wise cladograms (i‐v) correlates the closeness of patterns. Statistical association of the haplotypes in between patients with GBS and healthy controls was determined by odds ratios; *p*‐values were calculated from Fisher's exact test. *p*‐value < 0.05 was considered as significant (*).

We found 19 patterns of genotypes (haplotypes) with different frequencies (Fig. 2). Genotype‐based phylogeny generated three different clusters (A, B, and C) with three inclusions in each. The clade A consists of ‐592 C/C, ‐819 C/C and ‐1082 G/A genotypes; clade B includes ‐592 C/A, ‐819 T/T and ‐1082 G/G genotypes, and clade C clustered ‐592 A/A, ‐819 C/T and ‐1082 A/A genotypes together. These clusters signify greater chances of co‐occurrence of the genotypes existing under same clade. On column phylogram, closely related haplotypes formed five different clusters. Overall frequency distribution shows that cluster‐i (P3, P4, P6, P8, P9, and P11) and cluster‐ii (P1, P2, P13, and P20) occur more frequently (Fig. 2).

### Association of IL‐10 promotor polymorphisms and haplotypes with GBS susceptibility

The distribution of IL‐10 promoter polymorphisms (‐1082 G/A, ‐819 C/T and ‐592 C/A) and expression haplotypes in GBS patients with respect to healthy control individual were presented (Tables 2 and S1). The frequency of the homozygous genotype ‐819 TT was higher in patients of GBS compared to the healthy controls when comparisons were made prior to the Bonferroni correction of *p‐*values (*p* = 0.029, OR = 2.73, 95% CI = 1.15–6.45; *p*c = 0.08; Table 2) and the heterozygous −819 CT genotype was higher in healthy controls (*p* = 0.038, OR = 0.59, 95% CI = 0.37–0.96; *p*c = 0.114; Table 2). The ‐819 TT genotype was significantly prevalent in the axonal variant of GBS compared to healthy controls (*p* = 0.005, OR = 4.2, 95% CI = 1.55–11.40; *p*c = 0.015; Table 3) and the demyelinating subtype of GBS *(p* = 0.042, OR = 8.67, 95% CI = 1.03–72.97; Table 3). However, the *p‐*value lost its significance between axonal vs. demyelination following Bonferroni correction (*p*c = 0.123). The differences in the ‐1082 G/A and ‐592 C/A genotypes were not significant in accordance with GBS susceptibility (patient vs. control), *C. jejuni* LOS serology, anti‐GM1 antibody positivity, GBS subtypes, GBS severity, and outcomes (Tables 3, 4 and S2). Patients were categorized into different haplotype combinations including high (GCC/GTA, GCC/ATA and GCC/GCA; frequency ≥ 10.7%), medium (GCC/GCC, GCC/ACC, GCC/ACA, GCA/GTA, and GCA/GCA; frequency ≥ 2.7% and <10.6%), and low prevalence (rest of the haplotype combinations; frequency ≤ 2.6%) (Table S1). Haplotype analysis revealed no significant association between patients and healthy controls and presented distribution of major haplotype combinations as GCC/GTA (17.9% vs. 23.3%), GCC/ATA (10.6% vs. 15.3%), and GCC/GCA (13.9% vs. 10.5%), respectively, in the study cases and healthy controls (Fig. 2).

**Table 2:** Frequency distribution of IL‐10 promoter polymorphisms in patients with GBS and healthy controls.

Gene polymorphisms	GBS patients	Healthy control	*p‐*value	Odds ratio
	*n* = 152 (%)	*n* = 152 (%)		(95% CI)
‐1082(G/A)				
GG	103 (67.8)	97 (63.8)		Reference
GA	45 (29.7)	49 (32.2)	0.617	0.86 (0.53–1.41)
AA	4 (2.6)	6 (4.0)	0.532	0.63 (0.17–2.29)
‐819(C/T)				
CC	71 (46.7)	62 (40.8)		Reference
CT	56 (36.8)	82 (53.9)	*0.038^a^	0.59 (0.37–0.96)
TT	25 (16.5)	8 (5.3)	*0.029^b^	2.73 (1.15–6.45)
‐592(C/A)				
CC	35 (23)	37 (24.3)		Reference
CA	80 (52.6)	83 (54.6)	1.00	1.01 (0.58–1.78)
AA	37 (24.3)	32 (21.1)	0.614	1.22 (0.63–2.37)

GBS, Guillain–Barré syndrome; 95% CI, 95% confidence interval; *statistically significant.

a, *p*c = 0.114; b, *p*c = 0.08 (*p*c, *p* corrected).

**Table 3:** Distribution of IL‐10 promoter polymorphisms in axonal and demyelinating cases compared to healthy controls.

Genotypes	Axonal	Demyelinating	Healthy control	*p‐*value	Odds ratio	*p‐*value	Odds ratio	*p‐*value	Odds ratio
	*n* = 59 (%) (a)	*n* = 27 (%) (b)	*n* = 152 (%) (c)	a vs. b	(95% CI)	a vs. c	(95% CI)	b vs. c	(95% CI)
‐1082(G/A)									
GG	43 (72.9)	15 (55.6)	97 (63.8)		Reference		Reference		Reference
GA	15 (25.4)	12 (44.4)	49 (32.2)	0.132	0.44 (1.66–1.14)	0.319	0.69 (0.34–1.36)	0.282	1.58 (0.69–3.64)
AA	1 (1.7)	0	6 (4.0)	*nc*		0.675	0.38 (0.04–3.22)	*nc*	
‐819(C/T)									
CC	24 (40.7)	16 (59.3)	62 (40.8)		Reference		Reference		Reference
CT	22 (37.3)	10 (37)	82 (53.9)	0.47	1.47 (0.55–3.90)	0.309	0.69 (0.36–1.35)	0.091	0.47 (0.20–1.11.)
TT	13 (22)	1 (3.7)	8 (5.3)	*0.041^a^	8.67 (1.03–72.97)	*0.005^b^	4.2 (1.55–11.4)	0.682	0.48 (0.06–4.16)
‐592(C/A)									
CC	12 (20.3)	6 (22.2)	37 (24.3)		Reference		Reference		Reference
CA	30 (50.9)	17 (63)	83 (54.6)	1.0	0.88 (0.28–2.78)	0.847	1.11 (0.51–2.41)	0.805	1.26 (0.46–3.46)
AA	17 (28.8)	4 (14.8)	32 (21.1)	0.515	2.12 (0.49–9.2)	0.376	1.64 (0.68–3.94)	0.748	0.77 (0.20–2.97)

*nc*, not calculated; 95% CI, 95% confidence interval; a, *p*c = 0.123; b, *p*c = 0.015 (*p*c, *p* corrected); *statistically significant.

**Table 4:** Distribution of IL‐10 promoter polymorphisms and haplotype in severity of the disease and disease prognosis *in* patients with GBS.

Genotypes	Disease severity				GBS disability at 6 months			
	Severely affected	Mildly affected	*p*‐value	Odds ratio (95% CI)	Good outcome	Poor outcome	*p*‐value	Odds ratio (95% CI)
	*n* = 111 (%)	*n* = 41 (%)			*n =* 96 (%)	*n* = 56 (%)		
‐1082(G/A)								
GG	77 (69.4)	26 (63.4)		Reference	70 (72.9)	33 (58.9)		Reference
GA	30 (27)	15 (36.6)	0.324	1.48 (0.69–3.18)	25 (26)	20 (35.7)	0.192	1.7 (0.83–3.48)
AA	4 (3.6)	0 (0)	*nc*	*nc*	1 (1.1)	3 (5.4)	0.11	6.37 (0.64–63.55)
‐819(C/T)								
CC	51 (46)	20 (48.8)		Reference	44 (45.8)	27 (48.2)		Reference
CT	42 (37.8)	14 (34.1)	0.84	0.85 (0.38–1.89)	35 (36.5)	21 (37.5)	1.0	0.98 (0.47–2.02)
TT	18 (16.2)	7 (17.1)	1.0	0.99 (0.35–2.70)	17 (17.7)	8 (14.3)	0.637	0.77 (0.29–2.02)
‐592(C/A)								
CC	23 (20.7)	12 (29.3)		Reference	24 (25)	12 (21.4)		Reference
CA	63 (56.8)	17 (41.4)	0.164	0.55 (0.21–1.24)	49 (51)	30 (53.6)	0.681	1.22 (0.53–2.8)
AA	25 (22.5)	12 (29.3)	1.0	0.92 (0.34–2.45)	23 (24)	14 (25)	0.808	1.22 (0.47–3.18)

IL‐10 expression haplotype					IL‐10 expression haplotype			
	Mild form	Severe form	*p*‐value	Odds ratio (95% CI)	Independent locomotion	Unable to walk	*p*‐value	Odds ratio (95% CI)
	*n* = 41 (%)	*n* = 111 (%)			*n* = 96 (%)	*n* = 56 (%)		
High (frequency ≥ 10.7%)	11 (26.8)	53 (48.7)	*0.026	0.40 (0.19 to 0.90)	38 (39.6)	26 (46.4)	0.496	0.76 (0.40 to 1.43)
Medium (frequency ≥2.7‐ <10.6)	23 (56.1)	30 (31.5)	*0.001	3.45 (1.63–7.34)	33 (34.4)	17 (30.4)	0.721	1.20 (0.5998 to 2.40)
Low (frequency ≤ 2.6)	7 (17.1)	28 (19.8)	0.120	2.65 (0.98–8.22)	25 (26.0)	13 (23.2)	0.850	1.20 (0.56 to 2.85)

Patients at nadir with MRC‐sumscore < 40 were defined as severely affected patients and with MRC‐sumscore ≥ 40–60 were defined as mildly affected patients; ability to walk independently at 6 months of follow‐up was classified as good outcome (with GBS‐DS of 0, 1, and 2); unable to walk independently (with GBS‐DS of 3, 4, and 5) or death (with GBS‐DS of 6) as poor outcome^19^; GCC/GTA, GCC/ATA and GCC/GCA represent high frequency; GCC/GCC, GCC/ACC, GCC/ACA, GCA/GTA, and GCA/GCA represent medium frequency; frequency ≤ 2.6 represent low haplotype combinations; *statistically significant.

### Distribution of IL‐10 polymorphisms in clinical and serological subgroups of GBS

Genotype distribution of IL‐10 polymorphisms did not differ between the subgroups of patients with mild and severe form of GBS or among the patients with good outcome and poor outcome of the disease (Table 4). However, the haplotype distributions of these SNPs significantly differed between mildly and severely affected patients with GBS. The high IL‐10 expression (frequency ≥ 10.7%) haplotype combination GCC/GTA, GCC/ATA, and GCC/GCA was predominantly present (48.7% vs. 26.8%) in severely affected patients with GBS compared to mild form and reached statistical significance (*p* = 0.026, OR = 0.40, 95% CI = 0.19–0.90, *p*c = 0.078; Table 4). In addition, patients with severe form of GBS had significantly higher serum IL‐10 levels compared to the mild form of GBS (Mean [severely affected], 15.25 ± 51.72 pg/mL vs. mean [mildly affected], 3.59 ± 19.79 pg/mL, *p* = 0.046; Fig. 1B). The genotype frequencies of ‐1082 G/A, ‐819 C/T, and ‐592 C/A were analyzed to investigate the association between IL‐10 polymorphisms with *C. jejuni* infection and anti‐ganglioside antibody production (Table S2). The genotype distributions did not significantly differ between *C. jejuni* positive vs. *C. jejuni* negative GBS patients and anti‐GM1 antibody positive vs. anti‐GM1 antibody negative patients. The homozygous ‐819 TT genotypes were prevalent in *C. jejuni* serology positive patients compared to negative patients (21.1% vs. 8.8%) but the association was not significant (*p* = 0.088, OR = 0.36, 95% CI = 0.12–1.07; Table S2). Serum levels of IL‐10 were higher in patients that were anti‐GM1 antibody positive, *C. jejuni* LOS seropositive and patients with axonal variant of GBS (Fig. 1C–E). The high frequency expression haplotypes of IL‐10 were associated with serum IL‐10 in patients with GBS compared to healthy controls (*p* = 0.008, OR = 11.67, 95% CI = 1.86–128.7, *p*c = 0.024; Table 5).

**Table 5:** The association of IL‐10 expression haplotypes with serum IL‐10 in GBS and healthy controls and in severity.

IL‐10 expression	GBS [IL‐10(+)/*n*]	Healthy control [IL‐10(+)/*n*]	*p‐*value	Odds ratio (95% CI)	Mild form [IL10(+)/n]	Severe form [IL10(+)/n]	*p‐*value	Odds ratio (95% CI)
Haplotype								
High (frequency ≥ 10.7%)	10/64	1/64	*0.008	11.67 (1.86–128.7)	0/11	10/53	0.188	^#^0.18 (0.01–3.31)
Medium (frequency ≥2.7‐<10.6)	5/49	2/36	0.694	1.93 (0.37–10.11)	2/23	4/30	0.690	0.62 (0.11–2.90)
Low (frequency < 2.6)	5/38	1/35	0.201	5.15 (0.62–62.23)	0/7	4/28	0.562	^#^0.36 (0.02–7.55)

IL‐10(+), presence of serum IL‐10; n, number of subjects; Patients at nadir with MRC‐sum score < 40 were defined as severely affected patients and with MRC‐sum score ≥ 40–60 were defined as mildly affected patients; GCC/GTA, GCC/ATA, and GCC/GCA represent high frequency; GCC/GCC, GCC/ACC, GCC/ACA, GCA/GTA, and GCA/GCA represent medium frequency; frequency ≤ 2.6 represent low haplotype combination; “#,” Odds ratio was calculated by adding 0.5 to each value; *statistically significant.

## Discussion

We investigated the association of the three common polymorphic sites located in the promoter region of IL‐10 gene at ‐1082 G/A (rs1800896), −819 C/T (rs1800871), and ‐592 C/A (rs1800872) with the risk of developing GBS. This study indicates that the homozygous ‐819 TT genotype is associated with the axonal variant of GBS with respect to healthy controls and elevated IL‐10 expression haplotype combination GCC/GTA, GCC/ATA, and GCC/GCA may influence disease severity.

The homozygous ‐819 TT genotype was found to be prevalent in patients with GBS thus indicating a possible role in the susceptibility of GBS. However, after Bonferroni correction, this association was no longer significant yet indicating a trend of ‐819 TT genotype with GBS development. One of the previous studies claimed that the ‐592 CC and ‐819 CC genotypes are significantly predominant in Norwegian patients with GBS compared to controls.^18^ On the contrary, Geleijns et al. did not find any such association between Dutch GBS patients and healthy controls (Table 6).^22^ These incoherent findings could be an effect of the ethnic variation among the various populations involved in the association studies. Furthermore, we found that the ‐819 TT genotype was predominant in the axonal variant compared to the demyelinating form of GBS and/or the healthy individuals indicating a positive impact of this genotype with the axonal form. Previous studies also supported our findings, suggesting a correlation between increased IL‐10‐secreting blood mononuclear cells and axonal damage.^1^, ^23^ In addition, a strong influence of genetic factors on the production of IL‐10 was also described by Kasamatsu et al.^21^ However, Press et al. showed inconsistency with our findings showing high levels of pathogenic auto‐antibodies with increased IL‐10‐secreting blood mononuclear cells.^1^ Our study also supported the previous findings of Myhr et al. and Geleijns et al. which reported no associations of promoter polymorphisms with recent *C. jejuni* infections.^18^, ^22^ This study also confirms the previous findings of Nyati et al. with the increased expression of serum IL‐10 in GBS patients in the progressive phase of the disease compared with healthy controls.^32^ The significant upregulation of IL‐10 might be a result of restrain the production of pro‐inflammatory molecules to limit tissue damage and to maintain or restore tissue homeostasis in host^33^ indicating the potential anti‐inflammatory role of IL‐10 in GBS pathogenesis and severity.

**Table 6:** Association studies of IL‐10 promoter polymorphisms with GBS susceptibility.

Study (Author, year)	Ethnic origin/population	Country	Participants (*n*) (GBS vs. controls)	Methods	Major findings of IL‐10 promoter polymorphisms
Press, 2001 & 2002	Swedish/Caucasian	Sweden	41 vs. 55	ELISA	High levels of IL‐10‐secreting blood MNCs correlated with serum levels of anti‐ganglioside antibodies and axonal damage
Myhr, 2003	Caucasian	Norway	87 vs. 87	PCR	GBS patients had higher frequency of ‐592 CC and ‐819 CC genotypes compared to controls
Geleijns, 2007	Caucasian	Netherlands	263 vs. 210	PCR	No association of IL‐10 polymorphisms with disease susceptibility and severity
Current study	Asian	Bangladesh	152 vs. 152	PCR‐RFLP	‐819 TT genotype was prevalent in axonal variant compared to AIDP and healthy controls. Serum levels of IL‐10 was significantly higher in GBS compared to controls

ELISA, enzyme‐linked immunosorbent assay; IL‐10, Interleukin‐10; PCR‐RFLP, Polymerase chain reaction and restriction fragment length polymorphism.

Our study findings revealed a significant association of high frequency IL‐10 haplotype (GCC/GTA, GCC/ATA, and GCC/GCA) with disease severity. Moreover, we previously described that the ‐819 TT genotypes were prevalent in axonal variant of GBS which is the most severe form of GBS. The high IL‐10 expression haplotype combinations may somehow influence severe muscle weakness of patients since IL‐10 has pro‐inflammatory functions via B cell activation and inhibition of T cell apoptosis. Moreover, the polymorphisms of IL‐10 could affect the transcription, translation, and secretion of IL‐10.^34^ The production of IL‐10 is mainly controlled by the three studied polymorphisms of our research; however, the analysis of two additional IL‐10 polymorphisms at ‐1082 G/T (rs3024491) and ‐3575 T/A (rs1800890) was not performed and remains a limitation in our study.

In conclusion, the IL‐10 gene promoter polymorphisms ‐1082 G/A, ‐819 C/T and ‐592 C/A are not associated with susceptibility to GBS. However, homozygous −819 TT genotypes may have an impact on the axonal variant of GBS and high IL‐10 expression haplotype combinations (GCC/GTA, GCC/ATA, and GCC/GCA) may play a crucial role in disease severity. Large‐scale studies using a well‐designed cohort with populations of different ethnicities are required to confirm this relation and to get a clear understanding of the underlying genetic makeup concerning GBS pathogenesis.

## Author Contributions

Zhahirul Islam and Shoma Hayat conceived and designed the study. Shoma Hayat and Asaduzzaman Asad contributed to data acquisition. Shoma Hayat, Asaduzzaman Asad, Moriam Akter Munni, Md. Abu Jaher Nayeem, and Md. Golam Mostafa performed data analysis and interpreted the data. Zhahirul Islam and Shoma Hayat drafted the manuscript, which was critically reviewed by Asaduzzaman Asad, Moriam Akter Munni, Md. Abu Jaher Nayeem, Md. Golam Mostafa, Israt Jahan, Md. Zakir Hossain Howlader, and Quazi Deen Mohammad for intellectual content. All authors read and approved the final manuscript before submission.

## Conflict of Interest Statement

ZI received funding from the Fogarty International Center (FIC), National Institute of Neurological Disorders and Stroke of the National Institutes of Health (NIH), USA under Award Number K43 TW011447. SH received grant support from “Global Health Equity Scholars NIH FIC TW010540”, USA. SH, AA, MAM, MAJN, MGM, IJ, MZHH, QDM, and ZI have no conflicts of interest to declare.

## Supporting information


Table S1.

Table S2.
