# Supplementary material for: Interleukin‐10 promoter polymorphisms and haplotypes in patients with Guillain–Barré syndrome
Source: Ann Clin Transl Neurol. 2023 Nov 13;11(1):133–42. doi: 10.1002/acn3.51939 (PMC10791015; doi:10.1002/acn3.51939)
Supplement: Supplementary file 1 — Table S1. [file ACN3-11-133-s002.docx]

**Supplement Table 1**

**Table S1: IL-10 expression haplotype among patients with GBS and healthy controls**

| IL-10 expression  Haplotype | GBS  *n* = 152 (%) | Healthy control  *n* = 152 (%) | *P* value | Odds ratio  (95% CI) |
| --- | --- | --- | --- | --- |
| High  (frequency ≥ 10.7%) | 64 (42.1) | 74 (48.7) | 0.29 | 0.76 (0.49-1.2) |
| Medium  (frequency ≥ 2.7-< 10.6) | 50 (32.9) | 43 (28.3) | 0.45 | 1.24 (0.76-1.99) |
| Low  (frequency ≤ 2.6) | 38 (25.0) | 35 (23.0) | 0.79 | 1.11 (0.66-1.86) |

GBS, Guillain-Barré syndrome; GCC/GTA, GCC/ATA and GCC/GCA represent high frequency; GCC/GCC, GCC/ACC, GCC/ACA, GCA/GTA, and GCA/GCA represent medium frequency; frequency ≤ 2.6 represent low haplotype combinations.
