# Supplementary material for: Interleukin‐10 promoter polymorphisms and haplotypes in patients with Guillain–Barré syndrome
Source: Ann Clin Transl Neurol. 2023 Nov 13;11(1):133–42. doi: 10.1002/acn3.51939 (PMC10791015; doi:10.1002/acn3.51939)
Supplement: Supplementary file 2 — Table S2. [file ACN3-11-133-s001.docx]

**Supplement Table 2**

**Table S2: Distribution of IL-10 promoter polymorphisms in *C. jejuni* serology positive or negative patients and anti-GM1 antibody positive and negative patients with GBS**

| Genotypes | *C. jejuni* sero- positive  *n* = 95 (%) | *C. jejuni*  sero-  negative  *n* = 57 (%) | *P* value | Odds ratio  (95% CI) | *Anti-GM1-Ab* positive patients  *n* = 58 (%) | *Anti-GM1-Ab* negative patients  *n* = 94 (%) | *P* value | Odds ratio  (95% CI) |
| --- | --- | --- | --- | --- | --- | --- | --- | --- |
| -1082(G/A) |  |  |  |  |  |  |  |  |
| GG | 70 (73.7) | 33 (57.9) |  | Reference | 38 (65.5) | 65 (69.2) |  | Reference |
| GA | 25 (26.3) | 20 (35.1) | 0.192 | 1.7 (0.83-3.48) | 18 (31) | 27 (28.7) | 0.717 | 0.88 (0.43-1.78) |
| AA | 0 (0) | 4 (7) | *nc* | *nc* | 2 (3.5) | 2 (2.1) | 0.629 | 0.58 (0.08-4.32) |
| -819(C/T) |  |  |  |  |  |  |  |  |
| CC | 42 (44.2) | 29 (50.9) |  | Reference | 31 (53.4) | 40 (42.6) |  | Reference |
| CT | 33 (34.7) | 23 (40.3) | 1.0 | 1.0 (0.49-2.05) | 18 (31) | 38 (40.4) | 0.203 | 1.6 (0.79-3.4) |
| TT | 20 (21.1) | 5 (8.8) | 0.088 | 0.36 (0.12-1.07) | 9 (15.5) | 16 (17) | 0.638 | 1.38 (0.54-3.53) |
| -592(C/A) |  |  |  |  |  |  |  |  |
| CC | 24 (25.3) | 11 (19.3) |  | Reference | 14 (24.1) | 21 (22.3) |  | Reference |
| CA | 47 (49.4) | 33 (57.9) | 0.405 | 1.53 (0.66-3.55) | 28 (48.3) | 52 (55.4) | 0.676 | 1.24 (0.55-2.80) |
| AA | 24 (25.3) | 13 (22.8) | 0.806 | 1.18 (0.44-3.16) | 16 (27.6) | 21 (22.3 | 0.815 | 0.88 (0.34-2.24) |

*C. jejuni, Campylobacter jejuni;* 95% CI, 95% confidence interval; Anti-GM1-Ab, anti-GM1 antibody; 95% CI, 95% confidence interval.
